# The Instrumented Sit-to-Stand Test (iSTS) Has Greater Clinical Relevance than the Manually Recorded Sit-to-Stand Test in Older Adults

**DOI:** 10.1371/journal.pone.0157968

**Published:** 2016-07-08

**Authors:** Rob C. van Lummel, Stefan Walgaard, Andrea B. Maier, Erik Ainsworth, Peter J. Beek, Jaap H. van Dieën

**Affiliations:** 1 McRoberts BV, Raamweg 43, 2596 HN, The Hague, The Netherlands; 2 MOVE Research Institute Amsterdam, Department of Human Movement Sciences, Vrije Universiteit Amsterdam, Amsterdam, The Netherlands; 3 Department of Medicine and Aged Care, Royal Melbourne Hospital, University of Melbourne, Australia; Cardiff University, UNITED KINGDOM

## Abstract

**Background:**

The ability to rise from sitting to standing is critical to an individual’s quality of life, as it is a prerequisite for functional independence. The purpose of the current study was to examine the hypothesis that test durations as assessed with the instrumented repeated Sit-To-Stand (STS) show stronger associations with health status, functional status and daily physical activity of older adults than manually recorded test durations.

**Methods:**

In 63 older participants (mean age 83 ±6.9 years, 51 female), health status was assessed using the European Quality of Life questionnaire and functional status was assessed using the physical function index of the of the RAND-36. Physical performance was measured using a wearable sensor-based STS test. From this test, durations, sub-durations and kinematics of the STS movements were estimated and analysed. In addition, physical activity was measured for one week using an activity monitor and episodes of lying, sitting, standing and locomotion were identified. Associations between STS parameters with health status, functional status and daily physical activity were assessed.

**Results:**

The manually recorded STS times were not significantly associated with health status (p = 0.457) and functional status (p = 0.055), whereas the instrumented STS times were (both p = 0.009). The manually recorded STS durations showed a significant association to daily physical activity for mean sitting durations (p = 0.042), but not for mean standing durations (p = 0.230) and mean number of locomotion periods (p = 0.218). Furthermore, durations of the dynamic sit-to-stand phase of the instrumented STS showed more significant associations with health status, functional status and daily physical activity (all p = 0.001) than the static phases standing and sitting (p = 0.043–0.422).

**Conclusions:**

As hypothesized, instrumented STS durations were more strongly associated with participant health status, functional status and physical activity than manually recorded STS durations in older adults. Furthermore, instrumented STS allowed assessment of the dynamic phases of the test, which were likely more informative than the static sitting and standing phases.

## Introduction

The ability to rise from sitting to standing is a prerequisite for functional independence. Elderly who are unable to stand up from a chair without support are at risk of becoming more inactive and thus of further mobility impairment. The Sit-to-Stand (STS) transition is considered one of the most mechanically demanding physical activities in daily life [1]. Leg power has been associated with functional status [2] and functional ability [3]. Normal daily activities such as stair climbing and rising from a chair cause very high contact pressures in the human hip as measured in vivo [4]. STS transitions require the development of substantial muscle power [5] and consequently many older adults perform such transitions close to their maximal ability [6,7].

STS transitions are widely used as a test in clinical research and practice. The test is used either as stand-alone test or as part of the Short Physical Performance Battery (SPPB) [8]. Within the SPPB patients are invited to perform five STS transitions as quickly as possible with the time to perform these five repetitions being the test result. The SPPB has been shown to correlate with the amount of daily physical activity [9], the likelihood of future disability [10], the use of hospital services [11], nursing home admission [8] and mortality [12]. There is good evidence linking aging and COPD [13]. Also in pulmonary rehabilitation the use of the repeated STS [14] receives growing interest.

More detailed investigations of STS transitions, focusing on the nature of the dynamic STS phases, have been performed in laboratory settings using video-based 3D movement registration systems and force-plates [1,15,16]. However, such investigations are (too) time-consuming, complex and expensive for routine clinical usage. Inertial body fixed sensors provide an alternative approach to the laboratory to examine STS transitions in greater detail than manual STS recordings. This method has been used in this study and we call it the instrumented STS (iSTS). The present study was conducted to examine the merits of this alternative method, relative to the standard, hand-clocked STS test.

Several studies have shown that the durations and kinematic properties of the various STS phases can be successfully analysed using inertial body-fixed sensors [17,18]. Seat-off and seat-on detection in repeated sit-to-stand movements can be accomplished with sufficient accuracy for an objective measurement of task duration [19]. In a previous study, dynamic (standing up and sitting down) as well as static (standing and sitting) phases of the test could be determined [20]. Furthermore, in this study, age-related differences in STS performance were evident for all sub-phase durations. All STS phases (i.e., sit-to-stand, standing, stand-to-sit and sitting) were significantly longer and more variable in older compared to young adults [20]. In a small study (n = 11) it was shown that duration and variability of trunk movement during sit to stand could distinguish between elderly with high fall-risk and elderly with low fall-risk [21]. More recently, it has been suggested that parameters characterizing the rising phase of the STS cycle may be used to detect early frailty in clinical environments [22]. Indeed, several STS parameters showed significant differences between higher and lower functioning elderly as assessed by using a self-reported score of limitations in activities of daily living [23].

Compared to conventional manually recorded total test durations, fully automated analysis of repeated STS movements (e.g. durations, maximum angular velocity and angular displacement of STS sub-phases) may provide increased accuracy and ability to provide greater detail about the movement and hence may have added value.

The hypothesis of the current study was that test durations as assessed with the instrumented repeated STS show stronger associations with health status, functional status and daily physical activity of older adults than manually recorded test durations. To the best of our knowledge this is the first publication that investigated these associations.

## Methods

### Study population

Older participants were recruited from residential care facilities and the surrounding community. Eligible persons were aged 64 years and older, had a Mini-Mental State Examination (MMSE) [24] score > 18 out of 30 points, to include a wide range of cognitive abilities, and were able to walk 20 meter without cardiac or respiratory complaints. The medical ethical committee of the VU University Medical Centre Amsterdam approved the protocol for the study (#2010/290) and all participants provided written informed consent.

### Measures of participant characteristics

Participants were visited at home by a PhD student before the start of the project to explain the aim and procedure of the project, to collect baseline characteristics (age, gender, weight, height and body mass index) and cognition (MMSE) [24], and to ask the participant to sign informed consent.

### Measures of health status and functional status

Health status was assessed using the European Quality of Life questionnaire (EQ-5D-3L) [25]. This descriptive system comprises the following 5 dimensions: mobility, self-care, usual activities, pain/discomfort and anxiety/depression. A visual analogue scale records the respondent’s self-rated health. Functional status was assessed using the physical function index of the of the RAND-36 [26,27], which examines limitations in 10 activities related to mobility and physical movements.

### Physical performance

Physical performance was measured using the complete Short Physical Performance Battery (SPPB) protocol [10]. The SPPB is an objective assessment tool for evaluating lower extremity functioning in older persons and comprises measures of standing balance, walking speed, and ability to rise from a chair. For the chair stand participants were first asked to stand up from a straight-backed chair placed next to the wall, one time, without using their arms. If successful, participants were asked to rise from a chair with their arms crossed over their chest for five repetitions of standing up and four repetitions of sitting down, performed as fast as possible, and ending in a standing position. The manually recorded time was calculated as the duration of these 4.5 STS cycles. The 4 complete STS cycles were used for the instrumented analysis. The main reason to analyse 4 complete iSTS cycles instead of 4.5 STS cycles is technical in nature: drift correction of the raw signals is easier when the sensors end in the same position as they started. Furthermore, automatic detection of a complete STS cycle is more robust. Measuring the complete 4.5 STS made it also possible to automatically calculate the conventional STS sub-score of the SPPB.

The upper body makes the most significant contribution to both the vertical and the forward displacement of the centre of mass during standing up [1]. These upper body movements of the participants were measured using a small and light (87×45×14 mm, 74 grams) inertial sensor measurement system (DynaPort Hybrid, McRoberts, The Hague, The Netherlands). Acceleration and angular velocity were measured in three directions at a rate of 100 samples/s. The device was inserted in an elastic belt fixed around the waist near the spine over the undergarments and if possible beneath outer clothes. In this position it was unobtrusive, easy to fasten and least hampering the participant’s movements. This position near the centre of mass was chosen to measure whole body movements. The sensor location has been extensively used in geriatric settings [19,20,28] and the reliability of the measurements in a geriatric setting has been shown to be high [29].

The protocol for the test was implemented on a computer, which communicated with the measurement system via Bluetooth. The test leader used a remote control to send event markers to the protocol in the computer. The first marker was sent at `go`and the final marker was sent when the participant had straightened up completely for the fifth time. The assessor was standing close to the participant for reasons of safety. This manually recorded time was stored through the software. The signal analysis software automatically analysed the durations and the kinematic characteristics of the phases of the STS. This method, has been demonstrated to be valid [17,19,28] and reliable in a geriatric setting. ICCs were good to excellent for all variables in the total sample (0.80–0.94). The intra-observer group (50%) showed a higher number of excellent ICCs (≥.9) compared to the inter-observer subgroup (10%). SEM% was low for all variables (6.9–12.7%). The MDC_95_% ranged between 19.2–34.4% and more variables ≤30% were found in the intra- (80%) compared to the inter-observer group (60%) [29].

### Signal analysis

The measurement of 3-dimensional accelerations and angular velocities of the trunk allowed a detailed analysis of the different phases of the STS movement. Data were analysed using commercially available software (MoveTest, McRoberts, The Hague, The Netherlands).

**Fig 1:**
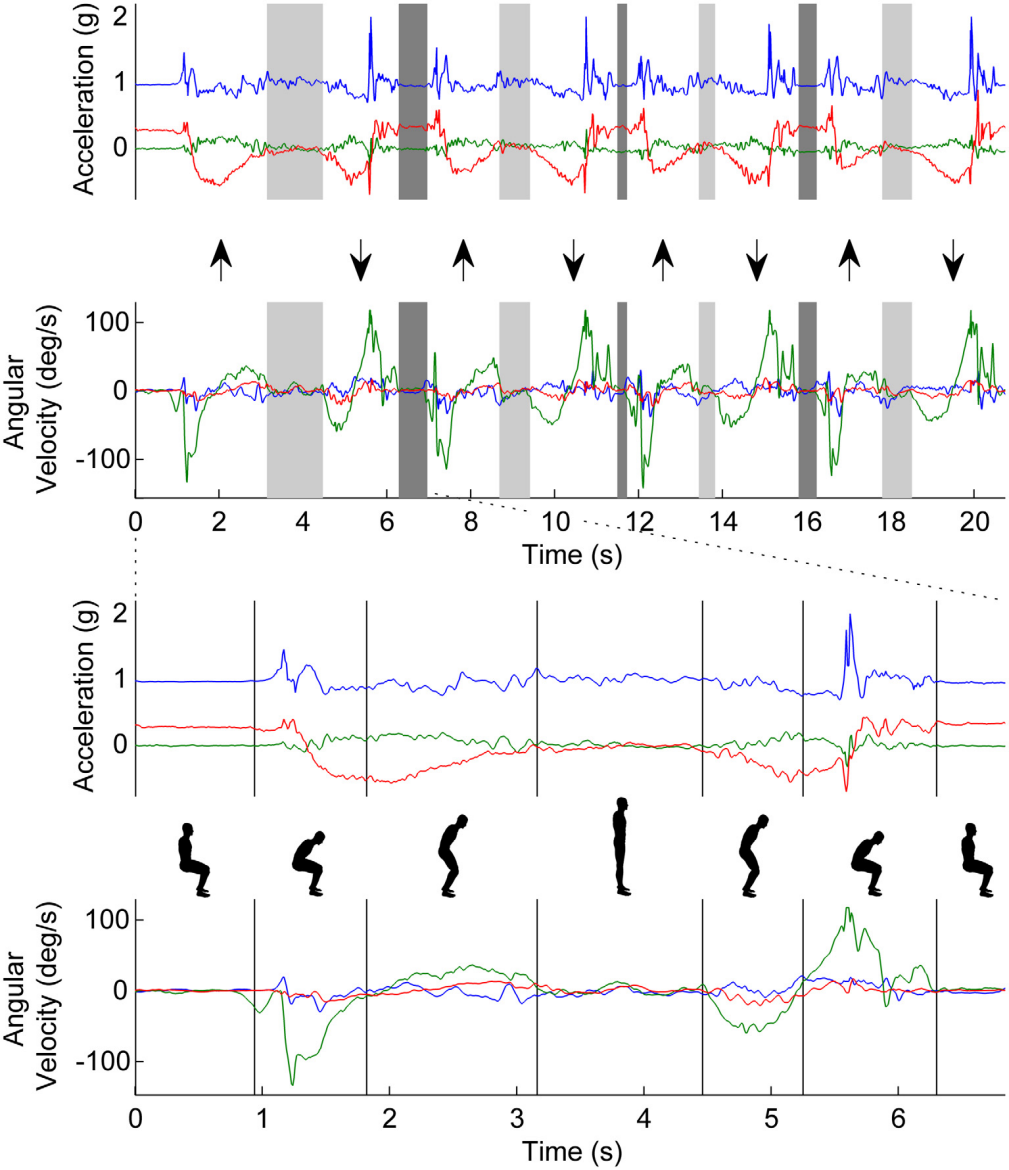
The top panel shows the time series of acceleration (green—mediolateral; red—anterior-posterior; and blue—vertical) and angular velocity (blue—pitch; green—yaw; and red—roll) over the main phases of the STS cycles. The ↑ arrows indicate standing up (SiSt) and the ↓ arrows indicate sitting down (StSi). The grey vertical bars demarcate the standing and sitting episodes. In the bottom panel the first complete STS cycle is depicted and magnified.

Fig 1 shows the filtered acceleration and angular velocity signals. In the upper panel, the up and down arrows indicate the standing up and sitting down phases, respectively. The sitting and standing phases were marked in grey.

The acceleration and the angular velocity were used to calculate the trunk angle [30] in the sagittal plane (flexion/extension). “True vertical acceleration” was estimated by removing the influence of the trunk angle from the vertical acceleration signal. Finally, vertical velocity was calculated by integrating this signal. This method has been described in more detail elsewhere [19]. Successful STS cycles were identified by an upward movement followed by a downward movement as identified by the vertical velocity. Drift and noise were removed from the trunk angle using discrete wavelet transform [21]. The local minima in this “cleaned up” signal were used to detect a change in trunk rotation direction. Each STS cycle contains 2 of such local minima which separate the flexion and extension phases of the trunk during sit-to-stand (SiSt) and Stand-to-Sit (StSi) (Fig 1, lower panel). The start of the sit-to-stand was defined as the end of the plateau before the first local minimum in the trunk angle. Similarly, the end of the sit-to-stand was defined as the start of the plateau after the first local minimum in the trunk angle. The start of the stand-to-sit was defined as the end of the plateau before the second local minimum in the trunk angle and the end of the of the stand-to-sit was defined as the start of the plateau after the second local minimum in the trunk angle. From the iSTS phases (SiSt, St, StSi and Si) mean durations, mean range of motion, mean maximum angular velocity and coefficient of variation (CoV) were calculated. CoV was expressed as the ratio of the standard deviation and the mean over the 4 repetitions times 100%. Only the durations and sub-durations were compared with the manually recorded time events in this study.

### Physical activity

Physical activity was measured using a small and light activity monitor (51×84×8.5 mm, 45 grams), which was attached centrally over the lower back with an elastic belt around the waist (DynaPort MM, McRoberts BV, The Hague, The Netherlands). Participants were asked to wear the activity monitor continuously for one week (i.e., 24/7) except during activities involving immersion of the body in water (e.g., when taking a shower). The monitor consisted of three orthogonal accelerometers (resolution: 0.003 g). Raw accelerometer signals were stored at a sampling rate of 100 samples/s. Reproducibility of the raw signals has been shown to be good to excellent. Intra- and inter-instrumental intraclass correlation coefficients (ICC) were all 0.99 and the intra-instrumental coefficients of variance were smaller than 1.13% [31].

The collected accelerometer data were analysed using commercially available software (MoveMonitor, McRoberts BV, The Hague, The Netherlands). First, the distribution of physical activity classes (lying, sitting, standing, locomotion, shuffling) and non-wearing was determined. Next, total duration, number of periods, and mean duration per period were calculated for these physical activity classes. The validity of such activity classifications has been demonstrated in both lab [32] and field [33,34] studies and one week of measurement has been shown to yield highly reliable results [35].

### Statistics

Continuous variables with a normal distribution were presented as mean and standard deviation (SD). If a skewed distribution (non-Gaussian) was found, the median and interquartile range (IQR) were determined. The STS durations were dichotomised, using a median split, in a slower and a faster performing group. These two groups were compared with regard to health status, functional status and daily physical activity (i.e., mean duration of sitting periods, mean standing duration and mean number of locomotion periods). Differences in outcomes between slow and fast performers were analysed using the Mann–Whitney U-test (SPSS Inc, Chicago, Illinois, USA). P-values below 0.05 were considered statistically significant.

## Results

Fifty-seven out of sixty-three older adults (mean age 84 years; SD ±11) produced complete data. Six participants were unable to complete the entire STS test and were excluded from the analysis. Four were unable to stand up with arms crossed and two were unable to finish the 5 repetitions. The mean duration of data collection for the SPPB (gait, balance and chair stand) was 6.5 minutes (SD 2.9 minutes). Total measuring time of the STS part of the SPPB exclusive putting on the equipment was 2.1 minutes. Mean time to prepare the STS was 1.2 (SD 0.86) minutes. Mean measurement time of the STS was 0.4 (SD 0.36) minutes. Removing the equipment took on average 0.3 (SD 0.36) minutes. These durations were collected during the study. In clinical practice, data collection might take more time. The duration of uploading and analyzing the data were not measured. Average wearing time of the activity monitor was 6.80 days with a minimum of 5.4 days. Mean wearing duration was 23.2 hours per day (96.7%).

The demographic, clinical and physical function parameters of the participants are shown in Table 1. The mean score for health status was 0.8 (±0.2). This was a bit higher than the normal scores as measured in the U.S. national health measurement study. People older than 74 years had a mean score of 0.7 [27]. The mean score for functional status was 57.3 (SD ±22.6), which is somewhat lower than measured in a clinical setting (36). In this study participants were younger (74 years ± 5.7) than in our study (84 years ± 11).

**Table 1:** Demographics, clinical characteristics, iSTS parameters and daily physical activity of the study population.

Characteristics				
(N = 57, 47 female, 25 care home)	Mean (SD)	Min	Max	Max/Min
**Demographics**				
Age (year)*	84.0 (11.0)	64.0	97.0	
Weight (kg)	73.6 (11.3)	50.0	98.8	
Height (m)	165.6 (7.9)	149.0	180.0	
BMI (kg/m2)	26.9 (4)	19.8	38.1	
**Clinical characteristics (points)**				
EQ-5D-3L (score)*	0.8 (0.2)	0.2	1.0	5.0
RAND-36 Physical function (score)	57.3 (22.6)	10.0	95.0	9.5
MMSE (score)*	28.0 (2.0)	20.0	30.0	1.5
**Manually recorded STS**				
4.5x STS duration (s)*	14.9 (6.6)	8.6	52.4	6.1
**Mean iSTS parameters (seconds)**				
SiSt duration*	1.7 (0.8)	1.0	3.1	3.1
SiSt flexion duration*	0.8 (0.2)	0.5	1.4	2.8
SiSt extension duration*	0.9 (0.4)	0.5	1.8	3.6
Stand duration	0.2 (0.5)	0	2.8	
StSi total duration*	1.7 (0.6)	1.0	3.4	3.4
StSi flexion duration*	0.8 (0.4)	0.5	2.0	4.0
StSi extension duration*	0.8 (0.3)	0.5	1.5	3.0
Sit duration	0.2 (0.6)	0	7	
**Daily physical activity**				
*Duration* #				
Lying duration (hr)	10.1 (2.1)	3.9	15.7	4.0
Sitting duration (hr)*	9.1 (2.7)	5.5	16.5	3.0
Standing duration (hr)	2.4 (0.9)	0.6	4.8	8.0
Locomotion duration (min)*	48.5 (30.4)	2.0	127.4	63.7
*Number of periods*				
Lying periods (N)*	8.5 (5.6)	1.5	38.0	25.3
Sitting periods (N)*	103.0 (36.2)	16.7	330.0	19.8
Standing periods (N)*	639.8 (399.6)	71.6	1488.6	20.8
Locomotion periods (N)*	297.3 (150.7)	15.6	769.7	49.3
*Mean duration of periods*				
Mean lying period duration (hr)*	1.3 (0.6)	0.3	3.8	12.7
Mean sitting period duration (min)*	5.7 (3.0)	1.9	28.7	15.1
Mean standing period duration (s)*	12.4 (4.0)	7.2	37.0	5.1
Mean locomotion period duration (s)*	9.4 (2.5)	5.8	16.2	2.8

Notes:

* Values are expressed as median (interquartile range)

^#^ Mean daily physical activity duration does not include non wearing and shuffling duration.

Shuffling is displacement during standing which has been been classified as locomotion.

Table 1 shows that the maximum for the STS mean sub-durations was 3 to 4 times as large as the minimum. The maximum durations of standing and locomotion in daily life were 9 to 64 times as large as the corresponding minimum durations. The maximum numbers of periods of standing and locomotion in daily life were 21 to 51 times as large as the corresponding minimum values. This indicates that extremes in outcomes differ less in physical performance (i.e. capability or capacity) than in physical activity (i.e. behavioural) outcomes.

**Fig 2:**
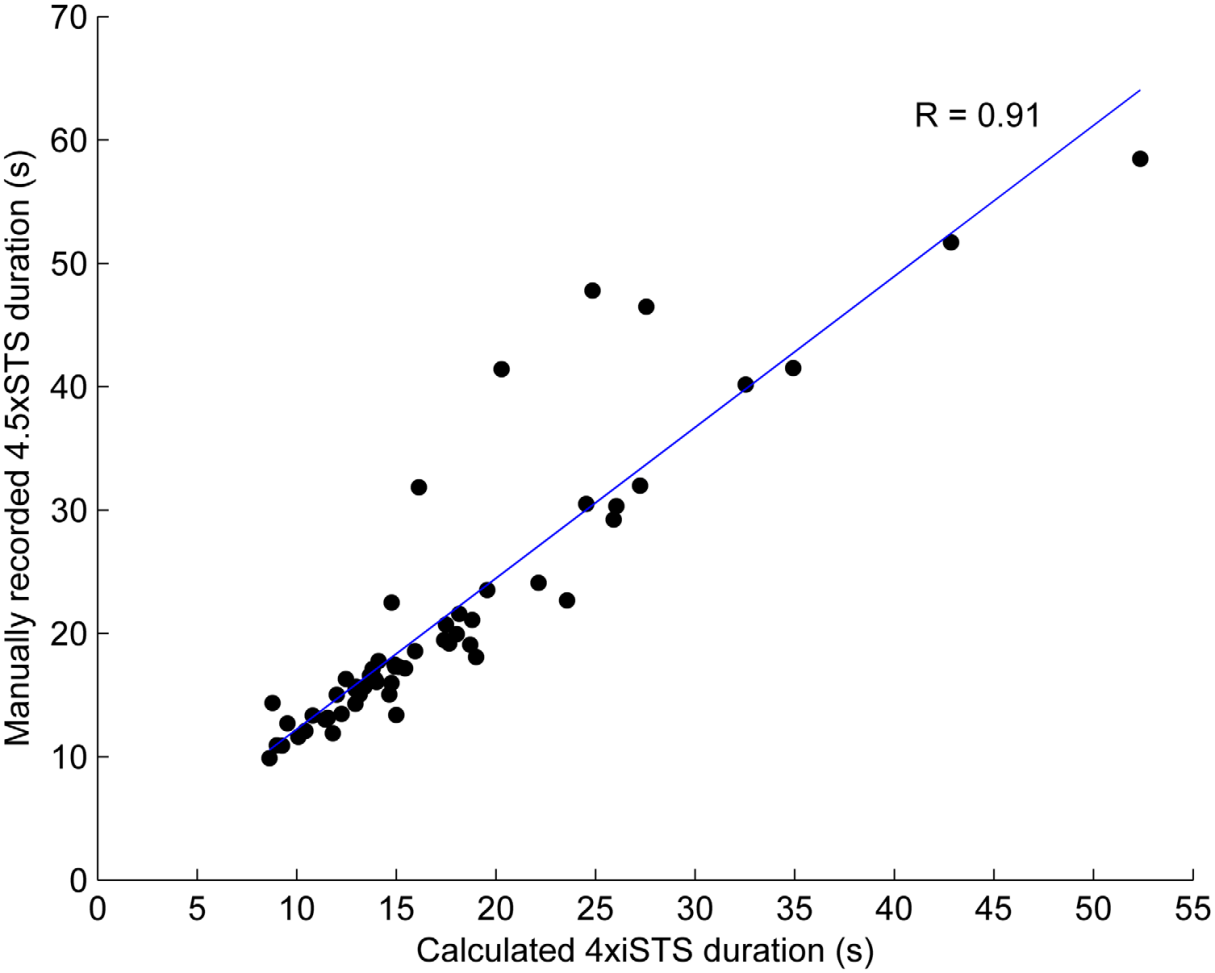
Associations between the durations (in seconds) of the manually recorded 4.5 x STS and the calculated 4 x iSTS.

Fig 2 shows the association between the manually recorded duration of the 4.5 STS and the duration of 4 STS as calculated using the instrumentation. All durations of the manually recorded data are longer because these include the 5^th^ SiSt. The four outliers had markedly longer manually recorded durations.

**Fig 3:**
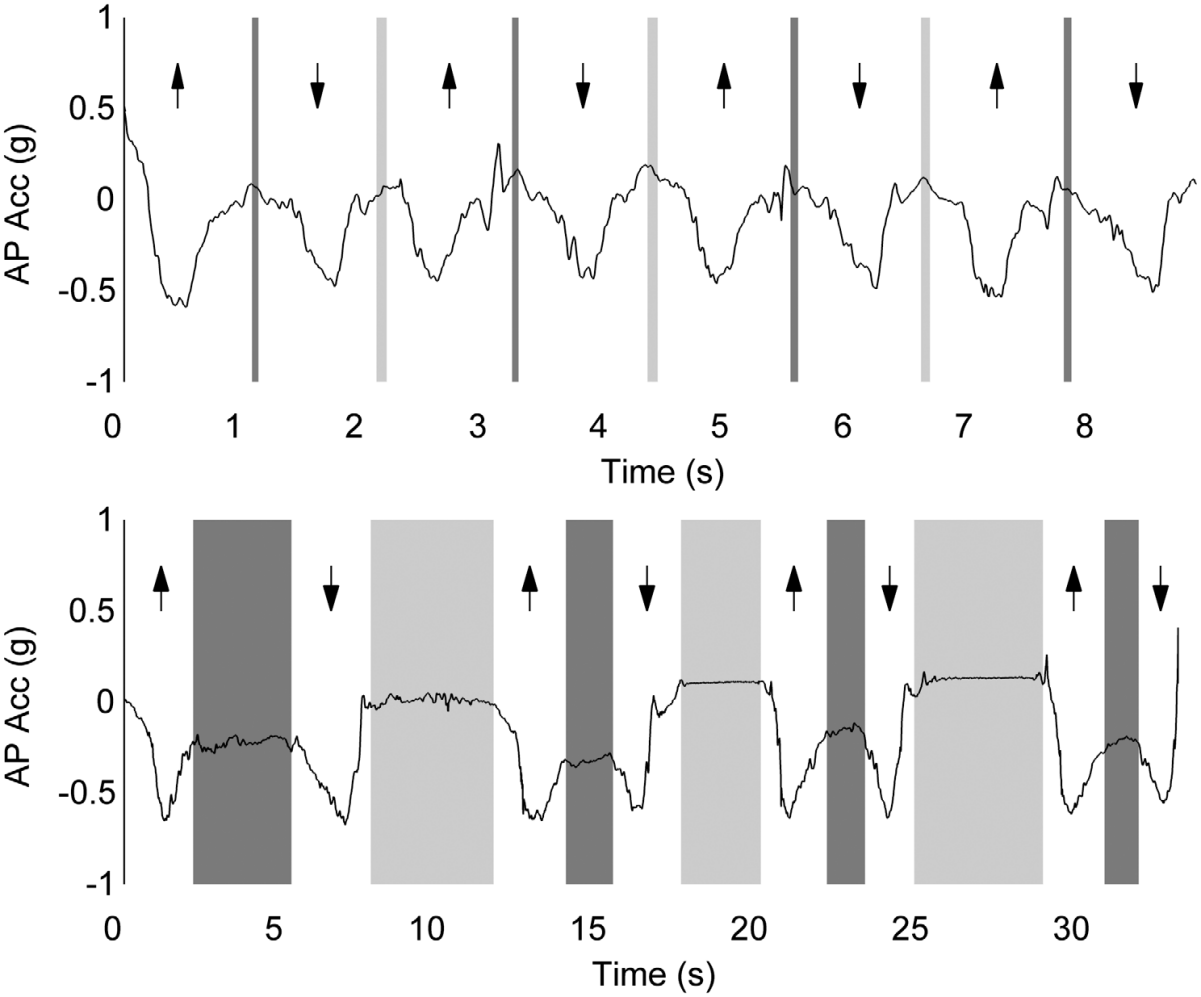
Anterior-posterior acceleration signal for two subjects. The ↑ arrows indicate standing up (SiSt) and the ↓ arrows indicate sitting down (StSi). The dark grey bars mark the standing duration, while the light grey bars mark the sitting duration. The upper panel shows a fast participant (9 seconds) with very short and regular standing and sitting durations. The lower panel shows a slow participant (33 seconds) with very long and less regular standing and sitting durations.

Fig 3 shows a typical example of a fast (upper panel) and a slow performer (lower panel) of the STS. The fast performer shows a regular pattern of durations with relatively short standing (dark grey) and sitting periods (light grey). The slow performer, in contrast, shows greater variation in durations and very long standing and sitting durations. Standing up and sitting down durations were respectively 1.9 and 1.8 times longer for the slow performer. Sitting and standing duration were respectively 36 and 50 times longer for the slow performer.

### The association of manually recorded and instrumented STS outcomes with health status and functional status

Table 2 shows the association of STS performance with health status (EuroQol) and functional status (RAND-36 Physical function). The manually recorded STS times were not significantly associated with health status (p = 0.457) or functional status (p = 0.055). In contrast, the 4 iSTS durations were associated highly significantly with health status and functional status (both p = 0.009). All the 6 SiSt parameters showed significant or highly significant associations with health status and functional status (p = 0.018–0.001). Two of the six StSi parameters showed significant associations with health status and functional status (p = 0.049 and p = 0.017).

**Table 2:** Associations of dichotomized STS and iSTS durations (seconds) by using a median split with health status (EuroQol) and functional status (RAND-36 Physical function). A higher number for the EuroQuol expressed a better health status. A higher number on the RAND-36 physical function expresses a better functional status.

		Health status			Functional status		
		EuroQuol 5D-3L			RAND-36 physical function		
		fast	slow	p-Value	fast	slow	p-Value
		performer			performer		
	**Manually recorded**						
↕	Duration 4.5xSTS	0.79	0.76	p = 0.457	64.8	51.2	p = 0.055
	**Movement duration**						
↕	Duration 4xiSTS	0.82	0.71	p = 0.009	65.9	47.5	p = 0.009
	**Sub-phase durations (mean of 4 STS cycli)**						
↗	SiSt duration	0.85	0.67	p = 0.001	67.7	44.2	p = 0.001
→	SiSt flexion duration	0.81	0.71	p = 0.015	64.4	47.5	p = 0.018
↑	SiSt extension duration	0.85	0.68	p = 0.001	68.5	44.5	p = 0.001
↔	Stance duration	0.80	0.75	p = 0.243	63.3	51.6	p = 0.097
↘	StSi duration	0.80	0.74	p = 0.237	63.7	50.0	p = 0.049
↓	StSi flexion duration	0.82	0.72	p = 0.017	63.0	51.4	p = 0.100
→	StSi extension duration	0.81	0.72	p = 0.150	60.2	53.7	p = 0.384
↔	Sit duration	0.79	0.75	p = 0.422	62.2	50.6	p = 0.091

↕ 4.5xSTS means 4 complete cycles and one SiSt ending in a standing position (SPPB)

↕ 4xSTS means 4 complete STS cycles, ending in a sitting position

↗ SiSt, including flexion and extension phase of standing up

→ SiSt, including flexion phase of standing up

↑ SiSt, including extension phase of standing up

↔ stance duration between standing up and before starting to sit down

↘ StSi, including flexion and extension phase of sitting down

↔ sit duration between sitting down and before standing up

### The association of manually recorded and instrumented STS outcomes with physical activity behaviour

Table 3 shows the associations between slow and fast STS performers (independent variable) with daily physical activity parameters (dependent variables). The faster performing group showed shorter mean duration of sitting periods, longer duration of standing and more locomotion periods. From the manually recordings durations only mean sitting period duration were significant. All movement duration of the 4 iSTS cycles showed highly significant differences between slow and fast performers (p = 0.001–0.002) for all physical activities. All nine SiSt associations showed highly significant associations with daily physical activity parameters. SiSt flexion and the extension durations showed significant associations (p = 0.001–0.010) with daily physical activity parameters. From the flexion and the extension duration during StSi only one of the 6 parameters showed a significant association with daily physical activity parameters.

**Table 3:** Associations between STS performance, dichotomized into fast and slow by a median split, with daily physical activity. Mean sitting period durations per day are expressed in seconds. Mean standing duration per day are expressed in minutes. Locomotion periods are expressed in mean number per day.

		Sitting			Standing			Locomotion		
		mean period duration (s)			duration of standing (m)			number of periods (n)		
		fast	slow	p-Value	fast	slow	p-Value	fast	slow	p-Value
		performer			performer			performer		
	**Manually recorded**									
↕	Duration 4.5xSTS	323	428	p = 0.042	156	139	p = 0.230	349	310	p = 0.218
	**Movement duration**									
↕	Duration 4xSTS	287	486	p < 0.001	169	123	p = 0.001	385	265	p = 0.002
	**Sub-phase durations (mean of 4 STS cycli)**									
↗	SiSt duration	286	487	p < 0.001	169	122	p = 0.001	387	263	p = 0.001
→	SiSt flexion duration	300	472	p = 0.003	166	126	p = 0.005	376	274	p = 0.010
↑	SiSt extension duration	297	474	p = 0.005	167	125	p = 0.003	386	264	p = 0.002
↔	Stance duration	332	439	p = 0.043	154	137	p = 0.200	349	302	p = 0.212
↘	StSi duration	304	468	p = 0.008	161	131	p = 0.040	368	282	p = 0.018
↓	StSi flexion duration	342	429	p = 0.218	152	140	p = 0.480	360	290	p = 0.106
→	StSi extension duration	322	449	p = 0.026	157	134	p = 0.109	361	289	p = 0.113
↔	Sit duration	324	447	p = 0.092	157	134	p = 0.218	356	295	p = 0.099

↕ 4.5xSTS means 4 complete cycles and one SiSt ending in a standing position (SPPB)

↕ 4xSTS means 4 complete STS cycles, ending in a sitting position

↗ SiSt, including flexion and extension phase of standing up

→ SiSt, including flexion phase of standing up

↑ SiSt, including extension phase of standing up

↔ stance duration between standing up and before starting to sit down

↘ StSi, including flexion and extension phase of sitting down

↔ sit duration between sitting down and before standing up

## Discussion

As expected, the associations with health status, functional status and physical activity between slow and fast STS performers were overall more significant for movement durations as determined with the iSTS than for manually recorded durations. The most plausible reason for this finding is that movement durations can be calculated more accurately when using iSTS than when recorded manually. The 4 outliers in the manually recorded durations may reflect such inaccuracies (see Fig 2). There might be a difference between the start signal of the test leader and the start of the movement because of different reaction times of the participants. The observed difference may also be related to the accuracy of the test leader, who has to mark the start and stop of the movement and simultaneously supervise the participant. In the present study, a third reason could be the difference between evaluating over 4.5 or 4 STS cycles. Observations of participants performing the test suggested that for some participants it was confusing to start in a sitting position and end in a standing position. They stopped after 4 cycles and had to be reminded to end in a standing position. Another reason might be the duration of the stabilization phase. In the official Short Physical Performance Battery Protocol and Score Sheet the end of the 5th StSi is when “he/she has straightened up completely for the fifth time” [36].” We used the raw signals to analyze the duration of the standing phase between the SiSt and the StSi. The variability of the duration expressed in the coefficient of variance of the standing phase has shown to be significant different comparing young and older adults [21]. This could be the fourth reason for the observed differences in duration between manual recording and instrumented detection.

A recent study aimed at determining the reliability of the instrumented timed up and go (iTUG) revealed no significant difference in reliability between manual recording and instrumented detection of total duration [36]. More research comparing manually and iSTS duration is necessary, especially for the shorter sub-durations.

Overall, the SiSt transition, which is performed against gravity, showed the strongest association with health status, functional status and daily physical activity. This might be related to the relatively old participants included in this study (median 84 years). The slower group on SiSt performance was significantly older (80.3 versus 85.6, p = 0.003).

Although we did not measure muscle mass, the corresponding degree of sarcopenia might also influence this outcome [38]. After all, it is estimated that after the 50^th^ year of life muscle mass and thus muscle force decrease with 1 to 2% per year, implying that the muscle mass of the participants was reduced considerably [39], which would limit their ability to stand up [6].

The difference between the associations of health status, functional status and PA with iSTS and STS revealed that the iSTS reflects more accurately the subject’s status than manually recorded STS durations, which are commonly used in clinical research and practice. These findings and insights provided by the associations are recapitulated in the following section along with their theoretical and practical implications.

### Associations between iSTS, health status and functional status

As already concluded, the iSTS durations showed stronger and more significant associations with self-reported health status and functional status than the manually recorded duration of the total test. Faster STS performers on the iSTS test exhibited higher scores for health status and functional status, which was not evident for the manually recorded durations. This difference could be due to the fact that clinically relevant information is mainly present in the dynamic phases of the STS (SiSt and StSi) and not in the static phases (St and Si), while the latter are included in the manually recorded time events but to a lesser extent in total iSTS and not in the durations of the dynamic phases. Slower performance of the complete STS cycle can be strongly influenced by longer durations of sitting and standing (Fig 3).

### Associations between iSTS outcomes and physical activity

As already concluded, the iSTS durations showed stronger and more significant associations with daily physical activity (mean duration of sitting periods, mean standing duration and mean number of locomotion periods) than the plain STS durations. Six of the seven iSTS parameters showed significant to strongly significant associations with the mean period durations of sitting measured in daily life. Faster performers showed shorter duration of sitting periods, longer standing durations and more locomotion periods. Recent studies have suggested that breaking up prolonged sitting may improve glucose metabolism and represent an important public health and clinical intervention strategy for reducing cardiovascular risk [40–43] and mortality [44].

Guralnik already stated in 1989: “Furthermore, performance tests may not give specific information on whether the identified limitations have any relevance to the actual activities or needs of the individual, or how well an individual with a limitation in a specific test item might have adapted to his or her individual environment (p. M143)” [45]. The activity monitor used in our study made it possible to compare in detail the physical performance outcomes with the individual’s physical activities in daily life because it provides detailed information about sedentary as well as active behaviour.

### Practical implications

Losing the ability to stand up without support has great implications for independent living. This is also evident in our data, which show clear associations between the ability to perform the STS and the amount and kind of daily activity. Therefore, in geriatric rehabilitation and physical activity programs, STS function should be considered as part of the training and the method discussed in this study may prove helpful for both diagnostic and evaluative purposes. This is in line with the plea of Guralnik [45] and Studenski [37] to include physical performance measures in the clinical setting. The instrumented STS test might be helpful for selecting appropriate and optimal interventions based on the patient’s physical performance profile and physical activity behavior and their associations [9].

We anticipate that future development will focus on the most important advantage of using body worn sensors, namely that they permit remote monitoring of ‘habitual’ STS behavior. This highlights the wider application of the findings of this study given that health status is related to the STS movement measured by a body worn sensor, the timing of which may be identified remotely and documented longitudinally.

It is also interesting to consider whether this association presented in the current study between STS times derived from body worn monitors and health status is retained when extracting STS repetitions from community ambulation data. The present data indicate that both self-perception of physical health and physical status are associated with, and potentially cause, slower and less successful STS performance, but also that these factors may affect the duration of STS phases differently. Previous studies have focussed specifically on the relation between the duration of repeated STS and knee muscle strength in terms of maximal force or power [6,46,47]. The development of muscle power is mainly required during the dynamic ascending phase of the STS transition. Further research should explore the associations of different phases of the iSTS with muscle strength and physical activity in daily life. The TENDO analyzer is an easy to use device that aims to measure power during the SiSt movement. This device might be used for such studies [48,49].

### Strength and limitations

iSTS may be readily applied in clinical settings. The single module instrumentation can be easily attached over undergarments and if possible beneath outer clothes in a manner that is unobtrusive to the subject. In this way the risk that the device is displaced is minimized. The awareness of being assessed is low because the instrumentation is not visible for the patient. Data collection is fast and with the remote control the test leader can stay close to the participant. The online connection of remote control makes it possible for one test leader to simultaneously collect data and watch over the participant as it is no longer necessary to read out the stopwatch and write down the times. The raw data are stored in the computer, which improves traceability and can be used for quality management. The automated analysis of the data provides detailed insight into the quality of the movements. The data are stored in a database, which makes it easy to use the clinical data for management and research purposes.

The high-resolution physical activity data, and consequently the ability to identify activity classes, provides more insight into health status, functional status and daily physical activity and its association with STS performance than using a single overall measure of acceleration.

The diversity of subjects is in general a positive aspect of the present study, with ages ranging from 66 to 97, BMI ranging from 20 to 38 and 44% recruited from residential care facilities. However, it is a concern that the number of subjects included in the analysis (N = 57) was relatively small. Although the present work represents a promising first step towards more detailed kinematic analyses of STS transitions, there is a clear need to collect reference data to compare sub-groups of older adults. Moreover, the present analysis focussed mainly on the duration of different STS phases. The range of motion, maximum angular velocity and the coordination between the different STS phases in terms of their relative timing have to be studied in greater detail in future studies, which are also needed to confirm the validity of the present findings and insights. A limitation of this study is that only cross-sectional data were collected. Future studies will have to reveal if the instrumented STS has added value in longitudinal and intervention projects.

The applicability of iSTS in a busy clinical environment remains to be demonstrated. Nevertheless, given its advantages and increased user-friendliness, we believe the method holds good prospects of finding wider application.

## Conclusions

Detailed outcomes of the instrumented STS were more strongly associated with health status, functional status and physical activity than manually recorded duration, and are thus likely to provide added value in clinical testing of older adults. Furthermore, iSTS revealed that the durations of the dynamic STS phase against gravity (SiSt) were markedly stronger associated with health status, functional status and daily physical activity than the total duration of the repeated STS. Participants with a better STS performance showed shorter mean sitting periods, longer mean standing durations and a higher mean number of locomotion periods in daily life, suggesting a more active lifestyle. Collectively, these findings suggest that a fully automated analysis of instrumented repeated STS movements may have greater clinical relevance compared to a manually recorded version of the test and may help to identify STS parameters that provide a basis for a more precise, quantitative studies of STS performance in clinical settings and clinical research. Fully-automated analyses means that the raw data collected during the STS measurement are uploaded to a webserver and analyzed automatically and that outcomes are stored in a database which can be used to generate reports.
